# Effect of Temperature on Magnetoimpedance Effect and Magnetic Properties of Fe- and Co-Rich Glass-Coated Microwires

**DOI:** 10.3390/ma18020287

**Published:** 2025-01-10

**Authors:** Paula Corte-Leon, Ivan Skorvanek, František Andrejka, Milos Jakubcin, Juan Maria Blanco, Valentina Zhukova, Arcady Zhukov

**Affiliations:** 1 Department of Polymers and Advanced Materials: Physics, Chemistry and Technology, Faculty of Chemistry, University of Basque Country, UPV/EHU, 20018 San Sebastian, Spain; paula.corte@ehu.eus (P.C.-L.); valentina.zhukova@ehu.es (V.Z.); 2 Department of Applied Physics I, Escuela de Ingeniería de Gipuzkoa EIG, University of Basque Country, UPV/EHU, Plaza Europa 1, 20018 San Sebastian, Spain; juanmaria.blanco@ehu.es; 3 EHU Quantum Center, University of the Basque Country, UPV/EHU, 20018 San Sebastian, Spain; 4 Department of Materials Science & Metallurgy, University of Cambridge, Cambridge CB3 0FS, UK; 5 Institute of Experimental Physics, Slovak Academy of Sciences, 040 01 Kosice, Slovakia; skorvi@saske.sk (I.S.); andrejka@saske.sk (F.A.); jakubcin@saske.sk (M.J.); 6 IKERBASQUE, Basque Foundation for Science, 48011 Bilbao, Spain

**Keywords:** giant magnetoimpedance, magnetic anisotropy, hysteresis loop, internal stresses, magnetic microwires

## Abstract

We provide new experimental studies of the temperature dependence of the giant magnetoimpedance (GMI) effect and hysteresis loops of Fe-rich and Co-rich amorphous microwires with rather different room temperature magnetic properties and GMI effect features. We observed a remarkable modification of hysteresis loops and magnetic field dependence of the GMI ratio upon heating in both of the studied samples. We observed a noticeable improvement in the GMI ratio and a change in hysteresis loops from rectangular to inclined upon heating in Fe-rich microwire. However, the opposite trend was observed in Co-rich microwire, in which, upon heating, the shape of the hysteresis loop changed from linear to rectangular. Generally, the evolution of the shape of the hysteresis loops during heating correlates with the modification of the dependencies of the GMI ratio Δ*Z*/*Z* on the magnetic field. For Co-rich microwire, the double-peak magnetic field dependence changed to single-peak, while for Fe-rich microwire, the opposite tendency was observed. The origin of the observed temperature dependences of the hysteresis loop and the GMI effect is discussed, considering internal stresses’ relaxation during heating, the temperature dependencies of the magnetostriction coefficient, and internal stresses, as well as the Hopkinson effect.

## 1. Introduction

Amorphous soft magnetic materials can present excellent magnetic and mechanical properties, making them suitable and promising for applications in several industrial sectors, such as magnetic and magnetoelastic sensors for airplane, car and space industries, electronic surveillance, energy harvesting, medicine, informatics, home entertainment, electrical engineering, magnetic recording, or magnetic memories, among others [1,2,3,4,5,6].

The quite unusual magnetic properties of amorphous wires, such as magnetic bistability associated with ultrafast magnetization switching by fast domain wall propagation or the giant magnetoimpedance, GMI, effect associated with excellent magnetic softness, have attracted enormous interest for technological applications [7,8,9,10,11]. Accordingly, amorphous magnetic wires have been intensively studied over the past three decades [6,7,8,9,10,11,12].

Glass-coated magnetic microwires fabricated using the Taylor–Ulitovsky technique have several relevant advantages, such as the most extended diameters range towards low diameters (between 0.5 and 100 µm), improved corrosion resistance, and biocompatibility [13,14,15]. Therefore, studies of this family of amorphous magnetic wires have attracted particular attention.

Excellent soft magnetic properties, a high GMI effect, ultrafast and controllable domain wall propagation, and magnetic bistability for the samples with a few mm length make these microwires suitable for several industrial applications in magnetic sensors and smart composites with tunable magnetic permittivity [16,17,18,19]. It is worth noting that the superior mechanical properties of amorphous glass-coated microwires with a reduced diameter allow for the development of the aforementioned technological applications [20,21].

Smart composites with magnetic microwire inclusions allowing external stimuli (magnetic field, applied stress, or heating) monitoring is among the most promising applications of glass-coated microwires [19,22,23,24,25]. The development of these microwave composites with magnetic wire inclusions is based on the dispersion of the effective permittivity ε_ef_ of such composites on the magnetic wires’ impedance and metallic wires’ geometry. The sensitivity of such composites with magnetic microwire inclusions on external stimuli originated by changing the wire magnetic properties upon the influence of such external stimuli (i.e., applied magnetic field, stress, or temperature) [19,26]. Particularly, the possibility of the in situ monitoring of matrix polymerization using magnetic microwire inclusions was recently demonstrated [24].

For the successful development of such applications, research into the influence of external stimuli on the GMI effect of magnetic microwires is essential. Although the influence of applied stress or magnetic field on the hysteresis loops and GMI effect of magnetic microwires has been widely studied [26,27,28,29,30], only a few studies have been conducted on the effect of temperature on the GMI effect [31,32,33,34,35]. Most studies on the effect of temperature on the GMI effect were performed in amorphous ribbons or in thick magnetic wires without glass coating and in a quite limited range of temperatures [29,30]. Very few studies on the temperature dependence of glass-coated microwires have been performed in a quite limited range of temperatures (below 100 °C) [33,34]. In some cases, heating was produced by an electrical current (which also produced a circular magnetic field) [34].

Recently, we studied the effect of heating on hysteresis loops and the GMI effect in Fe-rich glass-coated microwires in the temperature range from room temperature up to 300 °C and observed a substantial effect of temperature [35,36].

In this paper, we provide comparative studies of the temperature dependence of the GMI effect and hysteresis loops for Fe-rich (Fe_75_B_9_Si_12_C_4_) and Co-rich (Co_69.2_Fe_3.6_Ni_1_B_12.5_Si_11_Mo_1.5_C_1.2_) microwires, previously studied only at room temperature.

## 2. Materials and Methods

In this paper, we present comparative studies of temperature, *T* dependencies of hysteresis loops, and the GMI ratio of typical Fe-rich (Fe_75_B_9_Si_12_C_4_ with metallic nucleus diameter *d* = 15.2 µm and total diameter *D* = 17.2 µm) and Co-rich (Co_69.2_Fe_3.6_Ni_1_B_12.5_Si_11_Mo_1.5_C_1.2_ with *d* = 19.8 µm; *D* = 23.2 µm) glass-coated microwires prepared using the modified Taylor–Ulitovsky method described elsewhere [37,38].

**Figure 1:**
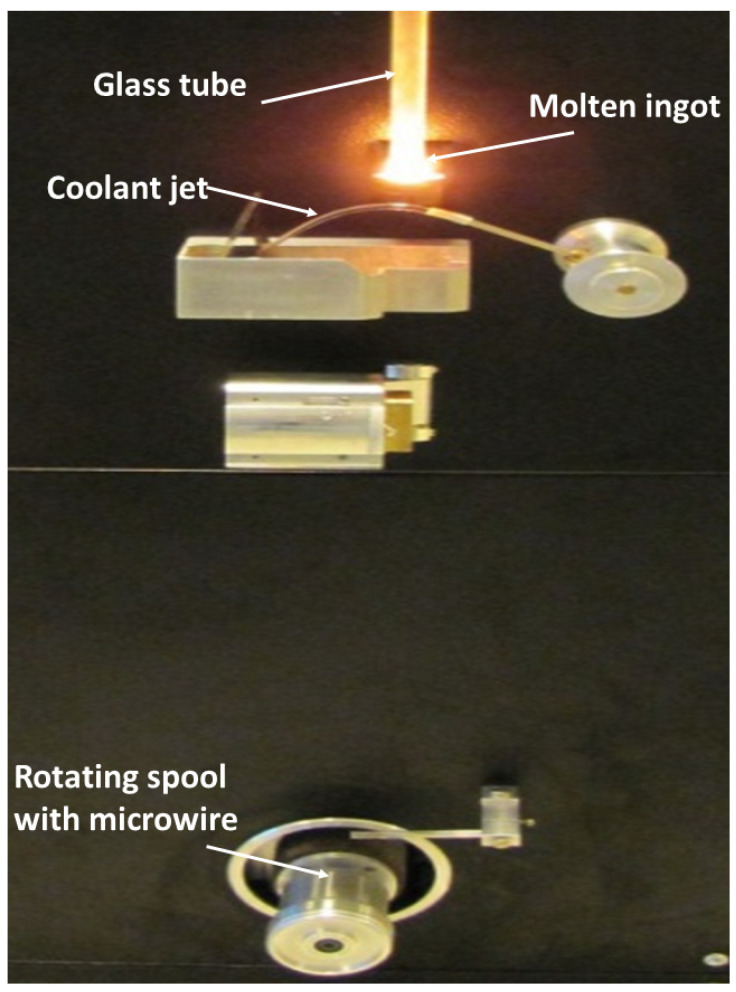
Sketch of the Taylor–Ulitovsky preparation process.

Essentially, the fabrication of glass-coated microwires involves melting a metal alloy ingot (typically a few grams) inside a glass (either Duran or Pyrex) tube using a high-frequency (usually 350–500 kHz) inductor heater, forming and drawing a glass capillary with the metallic alloy inside. The solidified glass-coated microwire is then captured by a rotating receiving bobbin. The manufacturing process sketch is shown in Figure 1. Rapid melt quenching, achieved by a coolant jet, allows us to obtain such glass-coated microwires with an amorphous structure [37,38].

The magnetostriction coefficients *λ_s_* of both of the studied microwires have been previously evaluated by the small-angle magnetization rotation (SAMR) technique [38]. The amorphous structure of both of the studied samples has previously been confirmed by X-ray diffraction (XRD) and differential scanning calorimetry (DSC) data [39]. The crystallization temperatures, measured by the DSC at a heating rate of 10 K/min, are 522 and 553 °C for Fe_75_B_9_Si_12_C_4_ and Co_69.2_Fe_3.6_Ni_1_B_12.5_Si_11_Mo_1.5_C_1.2_ microwires, respectively.

The hysteresis loops of both of the studied microwires were measured using the fluxmetric method and the vibrating sample magnetometer (VSM) MicroSense EV9. The VSM measurements were performed from room temperature up to 300 °C, as previously described elsewhere [35]. On the other hand, more precise hysteresis loop measurements of both of the studied samples have been performed using the fluxmetric method previously described in detail elsewhere [40]. All measurements were carried out with an axial orientation of the applied magnetic field. For a correct comparison of the magnetic properties of samples with different saturation magnetization, hysteresis loops were plotted as dependencies of the normalized magnetization *M*/*M*_0_ (*M*—the magnetic moment in a given magnetic field; *M*_0_—the magnetic moment of the microwire in the maximum magnetic field, measured at room temperature) on the applied magnetic field, *H*.

To evaluate the GMI effect, we used a specially designed experimental setup, which allows us to measure the GMI effect in a frequency range up to 110 MHz from room temperature to *T* = 300 °C [35]. The GMI effect is expressed as the GMI ratio, Δ*Z*/*Z*, determined as follows [7,11,38,41]:

Δ*Z*/*Z* = [*Z* (*H*) − *Z* (*H_max_*)]/*Z* (*H_max_*), (1)

with *Z*—the wire impedance and *H_max_*—maximum DC applied magnetic fields.

## 3. Experimental Results and Discussion

**Figure 2:**
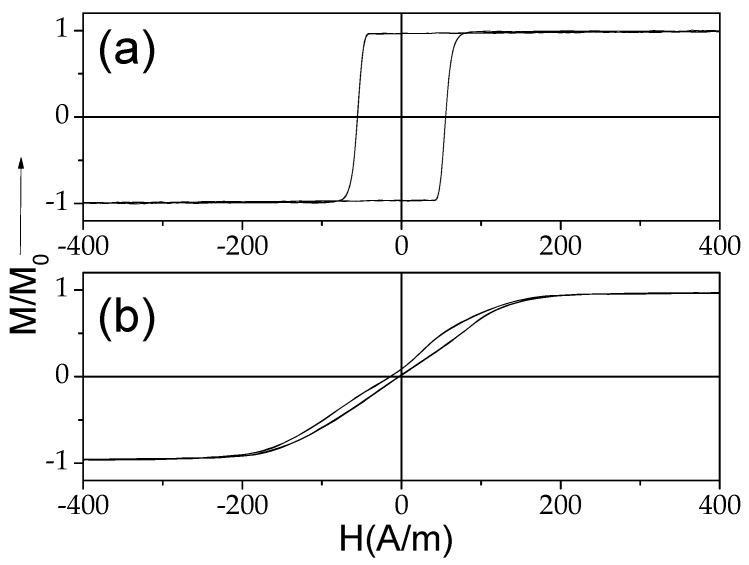
Hysteresis loops of as-prepared Fe_75_B_9_Si_12_C_4_ (**a**) and Co_69.2_Fe_3.6_Ni_1_B_12.5_Si_11_Mo_1.5_C_1.2_ (**b**) samples at room temperature.

The hysteresis loops of both of the studied samples measured at room temperature are provided in Figure 2. A rectangular hysteresis loop with coercivity, *H_c_*, about 60 A/m can be observed in the as-prepared Fe_75_B_9_Si_12_C_4_ sample (see Figure 2a), while an almost linear hysteresis loop with an order of magnitude lower than in the as-prepared Fe_75_B_9_Si_12_C_4_ sample *H_c_* (about 8 A/m) is observed in the Co_69.2_Fe_3.6_Ni_1_B_12.5_Si_11_Mo_1.5_C_1.2_ sample at room *T* (see Figure 2b). Such characters of hysteresis loops and *H_c_* values are typical for Fe-rich and Co-rich glass-coated microwires at room temperature with positive and vanishing *λ_s_* values [38].

The main source of the magnetic anisotropy of amorphous materials is the magnetoelastic anisotropy, directly affected by *λ_s_* and mechanical stress *σ*, consisting of internal stresses *σ_i_* and applied stresses *σ*_app_—(*σ*= *σ_i_* + *σ*_app_) [42]. The magnetoelastic anisotropy constant *K_me_* is given as follows [8,38]:

(2) $$K_{me}=3/2\lambda_{S}\sigma ,$$

Accordingly, the observed difference in the hysteresis loops must be attributed to different *λ_s_* values and sign.

Such different magnetic anisotropy of the studied microwires, axial for Fe-rich microwires with positive *λ_s_* and transverse for Co-rich microwires with low and negative *λ_s_*, is linked to the internal stress features. The simultaneous rapid quenching of the metallic alloy inside the glass coating is the main source of the internal stresses [29,37,38]. Generally, the character of such internal stresses is rather complex, but the axial internal stresses component is expected to be the largest within the most part of the volume of the metallic nucleus [29,37,39].

However, such internal stresses, induced by the difference in the thermal expansion coefficients of the metallic nucleus, *α_m_*, and glass coating, *a_g_*, are expected to be temperature (*T*)-dependent. The temperature dependence of *σ_i_* can be expressed as follows [33]:

(3) $$\sigma_{i}\approx E(\alpha_{g}-\alpha_{m})/\;T,$$

*E*—Young’s modulus.

Therefore, the modification of the magnetic anisotropy of glass-coated microwires is expected upon heating.

The experimental results on the temperature dependence of the magnetic properties and GMI effect are provided below.

### 3.1. Effect of Temperature on Magnetic Properties and GMI of Fe-Rich Amorphous Microwires

**Figure 3:**
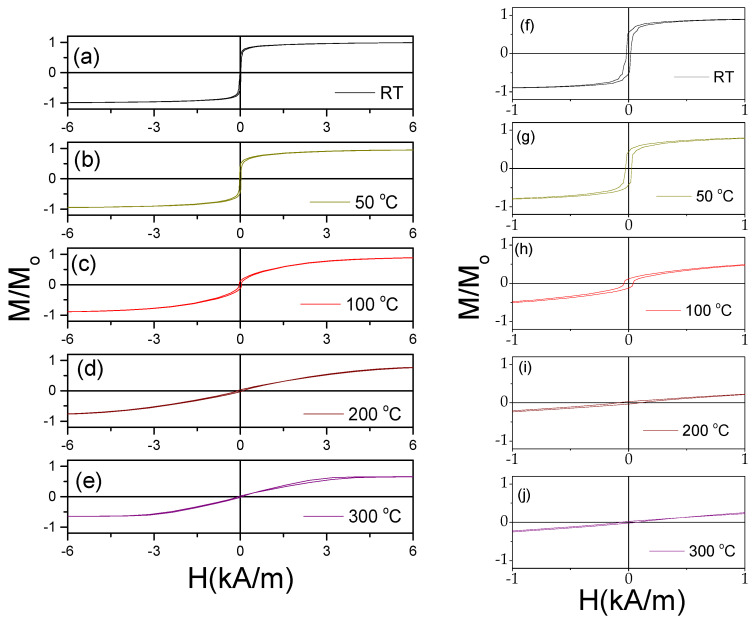

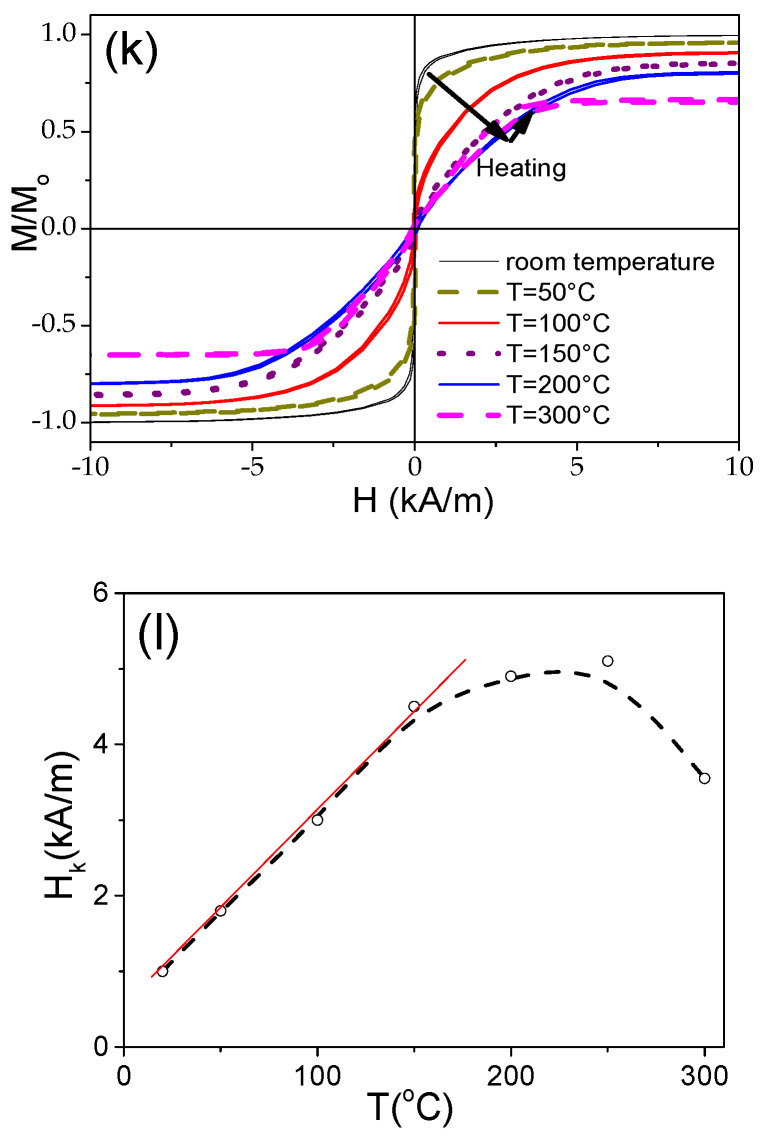
Hysteresis loops of Fe_75_B_9_Si_12_C_4_ sample, measured at different *T* (**a**–**e,k**), and low field hysteresis loops of the same sample (**f**–**j**) and *H_k_*(*T*) dependencies (**l**) evaluated from hysteresis loops. Figure (**l**) is adapted from [36,43]. Open Access Copyright © 2023 AIP and IARIA.

The temperature dependence of the hysteresis loops of Fe_75_B_9_Si_12_C_4_ is provided in Figure 3. A substantial hysteresis loops modification from rectangular into inclined upon heating is clearly seen (see Figure 3a–j). With increasing temperature, *T*, the hysteresis loop becomes more inclined. However, a change in this tendency is observed at *T* > 250 °C (see Figure 3d,e,k).

**Figure 4:**
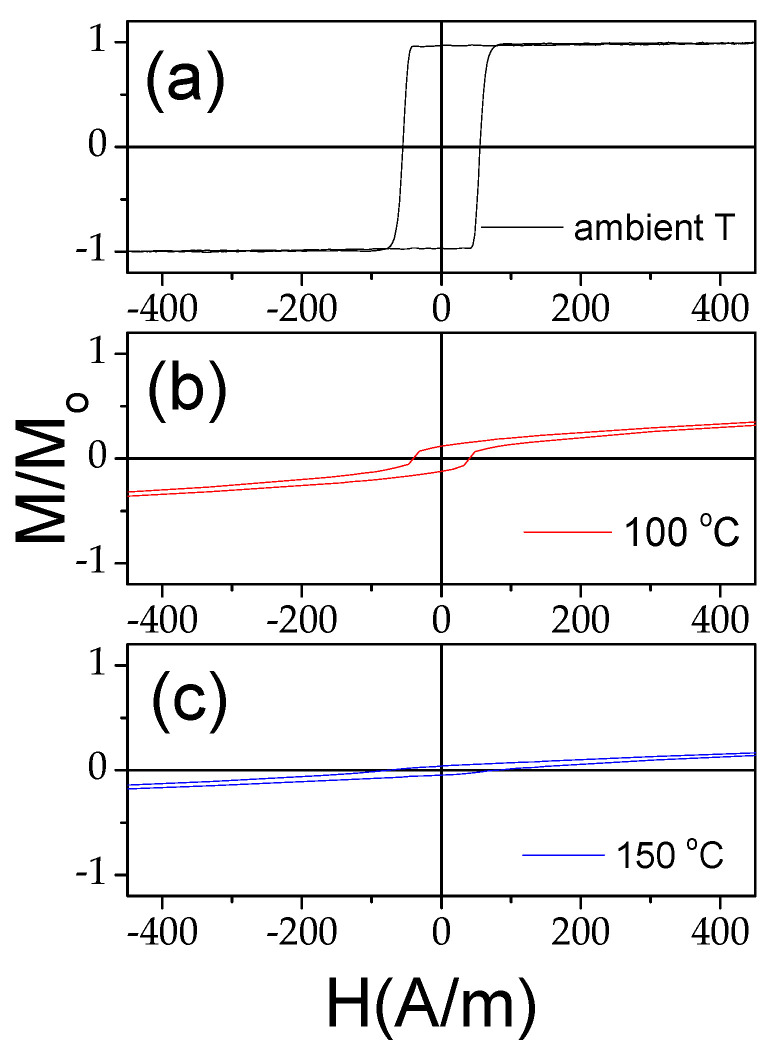
Hysteresis loops of Fe_75_B_9_Si_12_C_4_ sample measured at room *T* (**a**), at *T* = 100 °C (**b**), and at *T* = 150 °C (**c**).

This change can be better seen in Figure 3l, which shows the temperature dependence of the magnetic anisotropy field, *H_k_*, evaluated from the hysteresis loops. At low temperatures (*T* < 150 °C), the *H_k_*,(*T*) dependence appears linear. One of the reasons for the temperature dependence of *H_k_* must be attributed to the change in the internal stresses [43]. Indeed, the linear dependence of *H_k_* versus applied stress was previously reported for Co-rich microwires with vanishing negative *λ*_s_ [28]. Accordingly, the observed *H_k_*,(*T*) roughly correlates with Equation (3) for *T* ≤ 150 °C, while at *T* > 150 °C, the *H_k_*,(*T*) dependence becomes more complex. Such a deviation from the linear tendency in *H_k_*,(*T*) correlates with the change in the character of the hysteresis loops at *T* ≥ 150 °C. As can be observed from Figure 4, the characters of the hysteresis loops measured at room temperature and at *T* = 150 °C are rather different: the magnetic bistability related to the perfectly rectangular hysteresis loop disappears upon heating to 150 °C (see Figure 4a–c).

**Figure 5:**
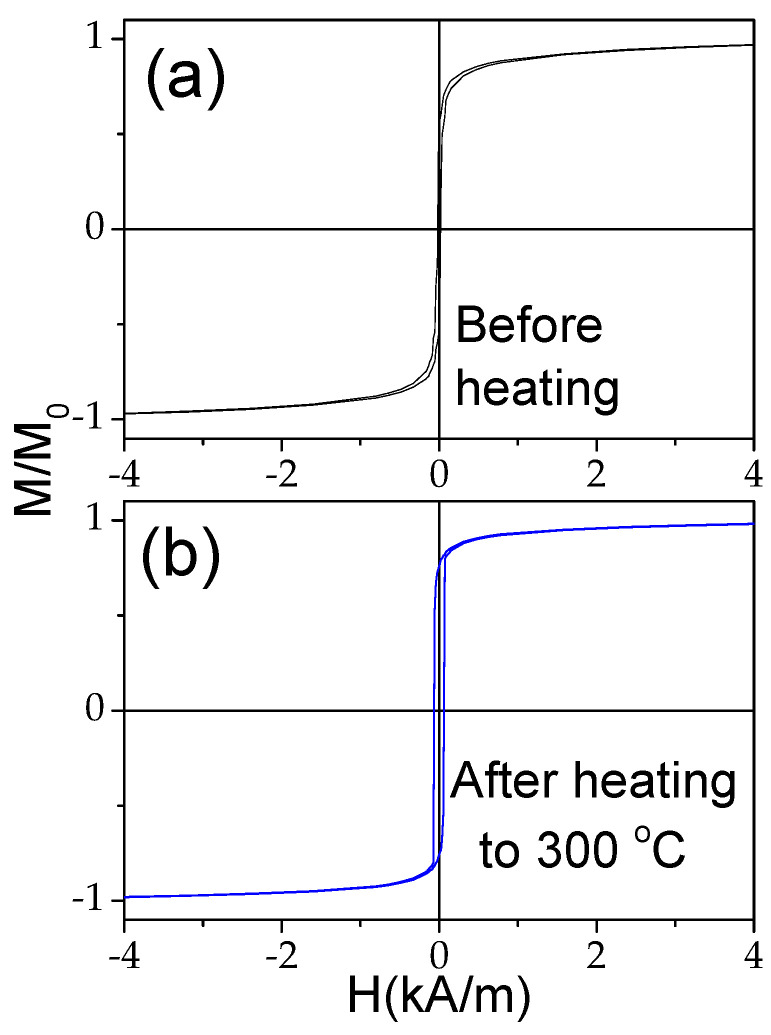
Hysteresis loops of Fe_75_B_9_Si_12_C_4_ sample measured before (**a**) and after heating up to 300 °C (**b**). Adapted from [36,43]. Open Access Copyright © 2023 AIP and IARIA.

On the other hand, the hysteresis loop measured after heating to 300 °C and cooling to room temperature restores its rectangular shape (see Figure 5). Accordingly, the observed changes in the hysteresis loops upon heating are almost completely reversible.

The origin of the GMI effect is satisfactorily explained from the point of view of the skin effect of magnetic conductors with high magnetic permeability [7,9,18]. As discussed elsewhere [9,11,44,45,46], the relationship between skin depth Δ and the circumferential magnetic permeability *μ_ϕ_* of magnetic wires is expressed as follows:

(4) $$\delta =\frac{1}{\sqrt{\pi \sigma \mu_{\phi }f}},$$

where *σ* is the electrical conductivity and *f* is the electrical current frequency.

Therefore, it can be assumed that the observed change in the hysteresis loops upon heating should be related to a modification of the GMI effect.

**Figure 6:**
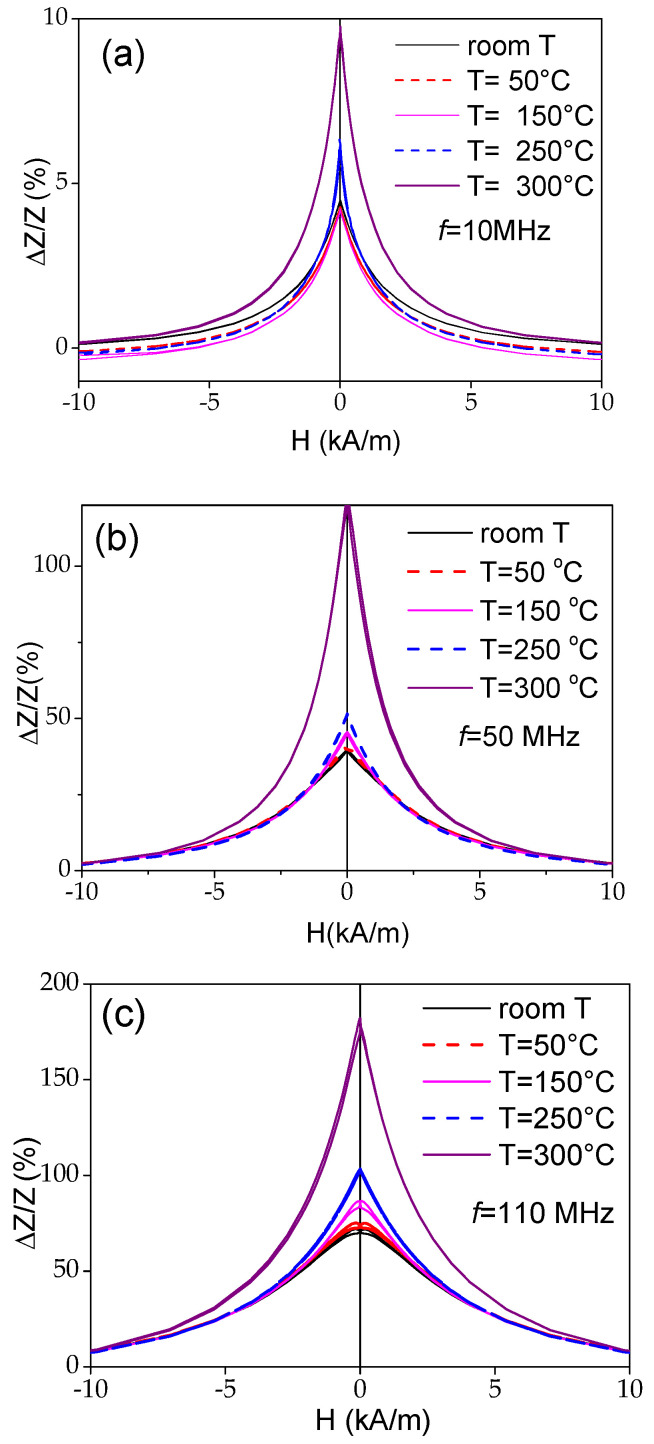
Δ*Z*/*Z*(*H*) dependencies of Fe_75_B_9_Si_12_C_4_ microwire measured at 10 (**a**), 50 MHz (**b**), and 1 and 100 MHz (**c**) at various temperatures.

The temperature dependence of the GMI effect of the Fe_75_B_9_Si_12_C_4_ microwire measured at different frequencies, *f*, is depicted in Figure 6. The main feature observed at all f is a remarkable increase in the GMI ratio at *T* = 300 °C. The maximum GMI ratio, Δ*Z*/*Z_max_*, of about 180% observed at *T* = 300 °C and *f* = 110 MHz is comparable to Δ*Z*/*Z_max_* reported at room temperature for Co-rich microwires elsewhere [45].

**Figure 7:**
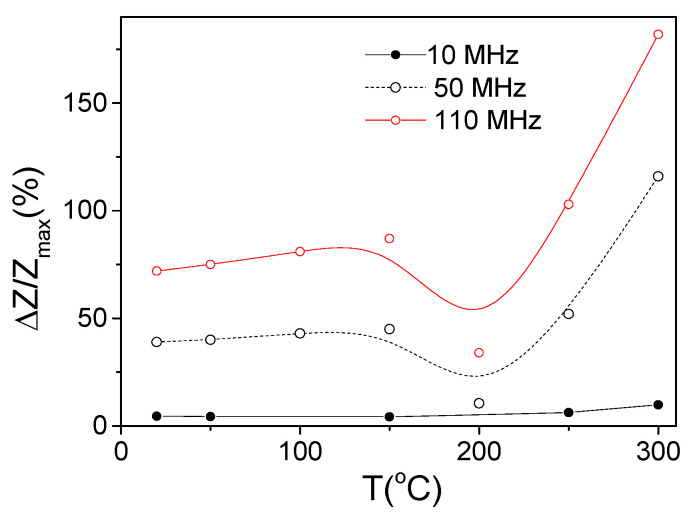
Δ*Z*/*Z_max_* (*T*) dependencies evaluated for 10, 50, and 110 MHz in Fe_75_B_9_Si_12_C_4_ sample.

The temperature dependencies of Δ*Z*/*Z_max_* evaluated from Δ*Z*/*Z*(*H*) dependencies measured at different *T* for 10, 50, and 110 MHz are summarized in Figure 7. From the Δ*Z*/*Z_max_*(*T*) dependencies, an increase in Δ*Z*/*Z_max_* at *T* ≥ 250 °C is clearly appreciated.

The observed increase in Δ*Z*/*Z_max_* upon the heating of Fe_75_B_9_Si_12_C_4_ microwire should be associated with several processes during heating.

One of such factors is the transformation of the hysteresis loops from rectangular into inclined upon the heating of the Fe_75_B_9_Si_12_C_4_ sample. Generally, it is assumed and experimentally demonstrated that the GMI effect is usually rather small in microwires with a rectangular character of hysteresis loops owing to low circumferential magnetic permeability [45]. The domain structure of such microwires exhibiting spontaneous magnetic bistability (with high positive *λ_s_*) is assumed to be consisting of an inner single domain magnetized axially and a radial magnetized outer domain shell [8,12,45]. As mentioned above, the axial character of the magnetic anisotropy of glass-coated microwires with positive *λ_s_* is attributed to the highest axial component of the internal stresses induced by the simultaneous rapid solidification of metallic nucleus and glass coating with rather different *α_m_* and *a_g_* values (see Equation (2)) [45,47,48,49]. As discussed above, the *σ_i_* values associated with the different *α_m_* and *a_g_* values are expected to decrease during heating.

The Hopkinson effect should also be involved in a noticeable increase in the GMI ratio observed at *T* = 300 °C. The origin of the Hopkinson effect is explained by considering a faster decrease in the magnetic anisotropy constant with temperature as compared to magnetization [50]. Therefore, a sharp maximum in magnetic permeability at temperatures slightly below the Curie temperature *T_c_* observed in magnetic materials is commonly explained by the Hopkinson effect [50,51]. Such interpretation looks reasonable considering a decrease in the magnetic anisotropy field at *T* ≥ 250 °C observed in *H_k_*(*T*) dependence (see Figure 3c).

Another factor that can influence the temperature dependence of the hysteresis loops and the GMI effect is the internal stresses’ relaxation associated with heating. As shown in Figure 5, the hysteresis loops measured at room temperature after heating to 300 °C remain almost unchanged. However, the skin depth at 100 MHz in magnetically soft microwires can be substantially smaller than the microwire diameter [52].

**Figure 8:**
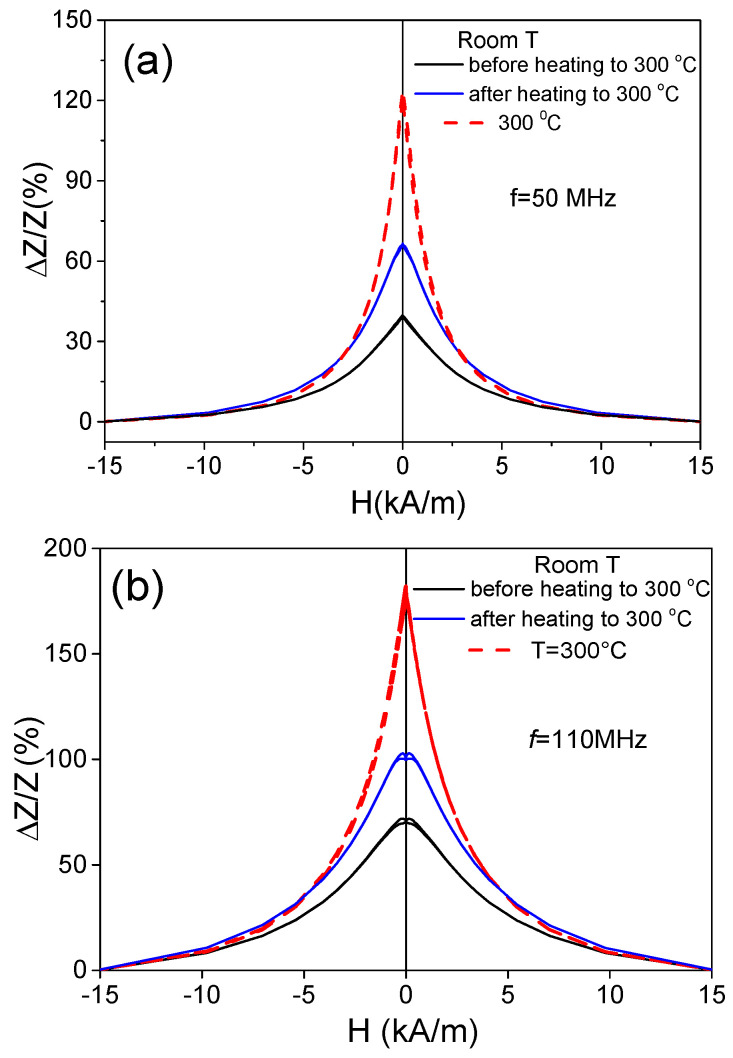
Δ*Z*/*Z*(*H*) dependencies measured at f= 50 MHz (**a**) and 110 MHz (**b**) at room temperature before and after heating at *T* = 300 °C in Fe_75_B_9_Si_12_C_4_ sample.

The attempt to separate the effect of heating from the effect of the internal stresses’ relaxation is provided in Figure 8, where the Δ*Z*/*Z*(*H*) dependencies measured at room temperature before and after heating up to 300 °C and at Δ*Z*/*Z*(*H*) dependencies measured at *T* = 300 °C are presented. Some increase in the Δ*Z*/*Z_max_* value at room temperature after heating to 300 °C is evident from the provided Δ*Z*/*Z*(*H*) dependences (see Figure 8). The observed difference in Δ*Z*/*Z_max_* at room temperature before and after heating to 300 °C must be associated with the relaxation of internal stresses. However, the main contribution to the increase in Δ*Z*/*Z_max_* is related to the heating itself, since the Δ*Z*/*Z_max_* values obtained at *T* = 300 °C are almost twice as high. This difference in Δ*Z*/*Z_max_* is observed at various frequencies (see Figure 8a,b).

Accordingly, the observed temperature dependence of the GMI effect and magnetic properties of Fe-rich microwires can be useful for temperature monitoring. However, the effect of heating must be separated from the internal stresses’ relaxation upon heating.

### 3.2. Effect of Heating on Magnetic Properties and GMI of Co-Rich Amorphous Microwires

As can be appreciated from Figure 2b, Co_69.2_Fe_3.6_Ni_1_B_12.5_Si_11_Mo_1.5_C_1.2_ microwires present rather good magnetic softness with coercivity, *H_c_*, of about 4 A/m and a magnetic anisotropy field, *H_k_*, of about 180 A/m at room temperature.

**Figure 9:**
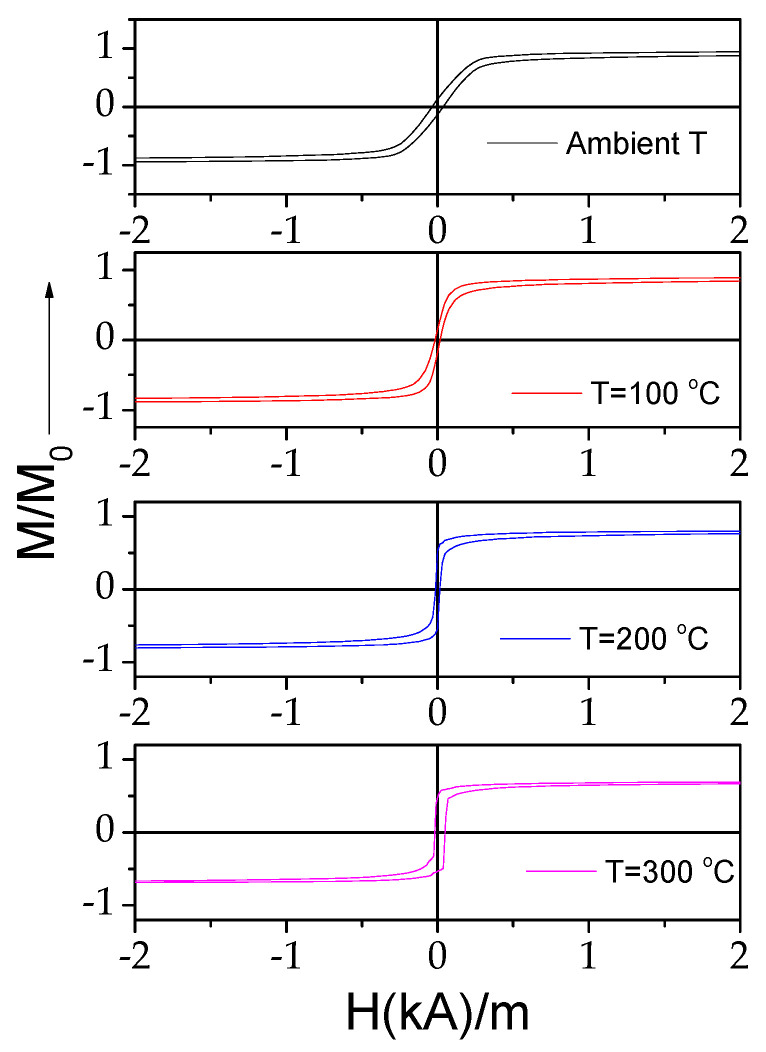
Effect of heating on hysteresis loops of Co_69.2_Fe_3.6_Ni_1_B_12.5_Si_11_Mo_1.5_C_1.2_ microwires.

Similarly to Fe-rich microwires, in Co_69.2_Fe_3.6_Ni_1_B_12.5_Si_11_Mo_1.5_C_1.2_ microwires upon heating, a significant change in the hysteresis loops is observed (see Figure 9): the shape of the hysteresis loops changes from almost linear to nearly rectangular. The observed modification in the hysteresis loop character is directly opposite to that observed in the Fe_75_B_9_Si_12_C_4_ microwire.

The origin of the observed modification in hysteresis loop shape upon heating can be understood considering the similar transformation of linear hysteresis loops into rectangular upon the annealing of Co-rich microwires with nearly zero *λ_s_* [39,46,52]. The origin of such a transformation in the shape of hysteresis loops from linear to rectangular is explained in terms of the change in the *λ_s_* value and sign due to internal stresses’ relaxation [39,46,52].

**Figure 10:**
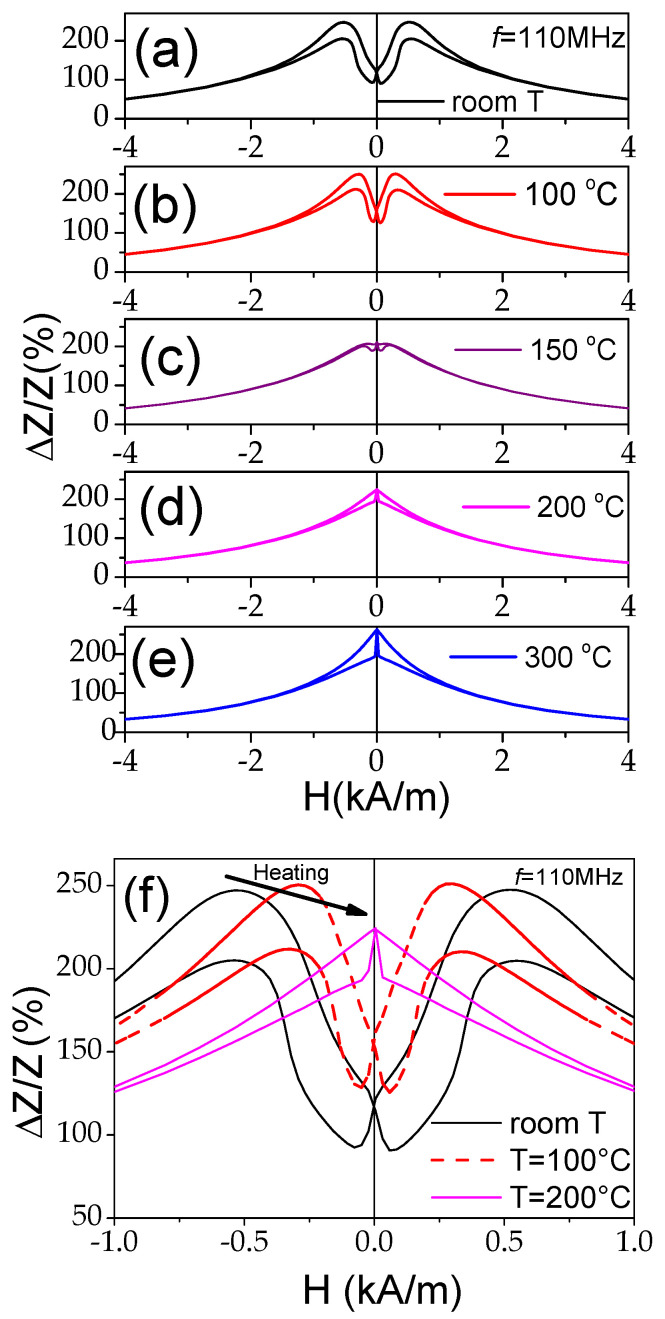
Δ*Z*/*Z*(*H*) dependencies measured at *f* = 110 MHz at room temperature (**a**), 100 °C (**b**), 150 °C (**c**), 200 °C (**d**), and 300 °C (**e**) and modification of the Δ*Z*/*Z*(*H*) dependencies measured at 110 MHz upon heating of Co_69.2_Fe_3.6_Ni_1_B_12.5_Si_11_Mo_1.5_C_1.2_ microwires (**f**).

**Figure 11:**
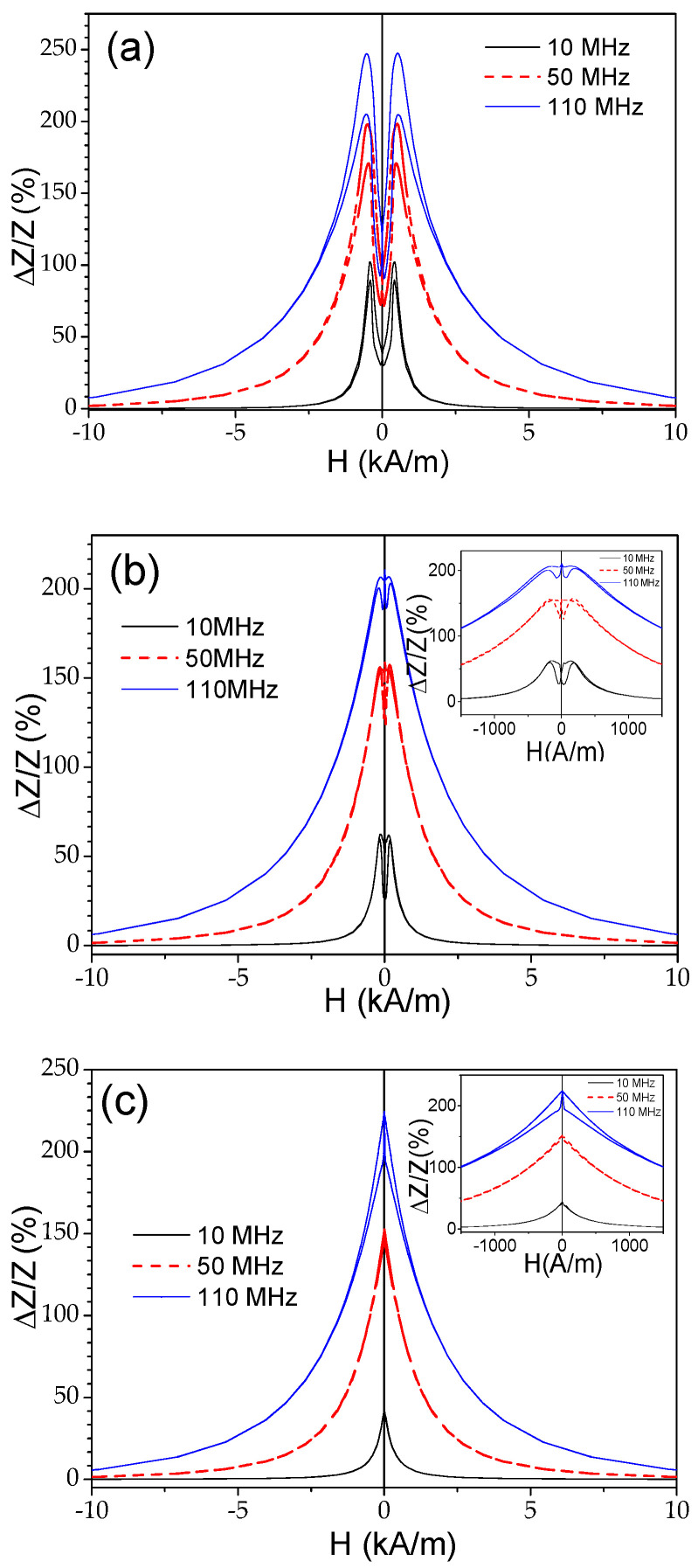
Δ*Z*/*Z*(*H*) dependencies of Co_69.2_Fe_3.6_Ni_1_B_12.5_Si_11_Mo_1.5_C_1.2_ microwires measured at room temperature (**a**), at 150 °C (**b**), and at T= 200 °C (**c**).

As discussed elsewhere [45,53], the Δ*Z*/*Z* magnitude and shape of Δ*Z*/*Z*(*H*) dependencies are related to the magnetic anisotropy of the magnetic wires. Therefore, one can expect changes in the Δ*Z*/*Z* value and Δ*Z*/*Z*(*H*) dependencies upon heating. As expected, the observed change in the magnetic anisotropy of Co_69.2_Fe_3.6_Ni_1_B_12.5_Si_11_Mo_1.5_C_1.2_ microwires correlates with the changes in the Δ*Z*/*Z* value and Δ*Z*/*Z*(*H*) dependencies. The effect of temperature on the Δ*Z*/*Z*(*H*) dependencies can be observed in Figure 10, where the Δ*Z*/*Z*(*H*) dependencies measured at different temperatures *T* are provided. As can be appreciated from Figure 10 and Figure 11, a gradual change from double-peak to single-peak Δ*Z*/*Z*(*H*) dependence takes place upon heating at 150 ≤ *T* ≤ 200 °C (see Figure 10). Thus, at *T* = 150 °C, a double peak Δ*Z*/*Z*(*H*) dependence can still be observed for all the frequencies (see Figure 11b). However, as compared to the Δ*Z*/*Z*(*H*) dependence measured at room *T*, the magnetic field of Δ*Z*/*Z*(*H*) maximum *H_m_* becomes smaller. Finally, at *T* ≥ 200 °C, single-peak Δ*Z*/*Z*(*H*) dependencies are observed for all *f* values (see Figure 11c).

The aforementioned field of maximum *H_m_* on Δ*Z*/*Z*(*H*) dependencies is attributed to the magnetic anisotropy field at MHz frequencies [45,53]. Thus, single-peak Δ*Z*/*Z*(*H*) dependencies correspond to the case of magnetic wires with axial magnetic anisotropy [45,53]. Accordingly, the observed modification in Δ*Z*/*Z*(*H*) dependencies upon heating correlates with the temperature dependence of the hysteresis loops, shown in Figure 9. Such changes must be attributed to the change in the magnetic anisotropy from weak transverse magnetic anisotropy to axial magnetic anisotropy upon heating.

**Figure 12:**
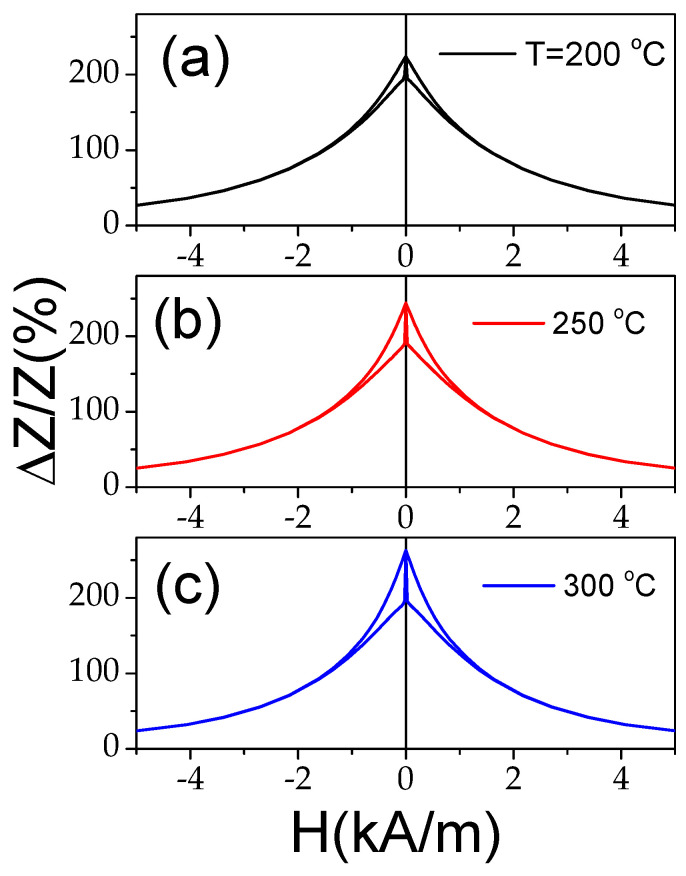
Δ*Z*/*Z*(*H*) dependencies of Co_69.2_Fe_3.6_Ni_1_B_12.5_Si_11_Mo_1.5_C_1.2_ sample measured at f = 110 MHz at *T* = 200 °C (**a**), *T* = 250 °C (**b**), and T= 300 °C (**c**).

As can be seen from Figure 11, despite the axial character of the hysteresis loop upon heating, the Δ*Z*/*Z_max_* values remain relatively high. However, from a comparison of the Δ*Z*/*Z*(*H*) dependencies measured in Co-rich samples with the same character of hysteresis loops (see Figure 12), some increase in the Δ*Z*/*Z_max_* values is visible with increasing T.

**Figure 13:**
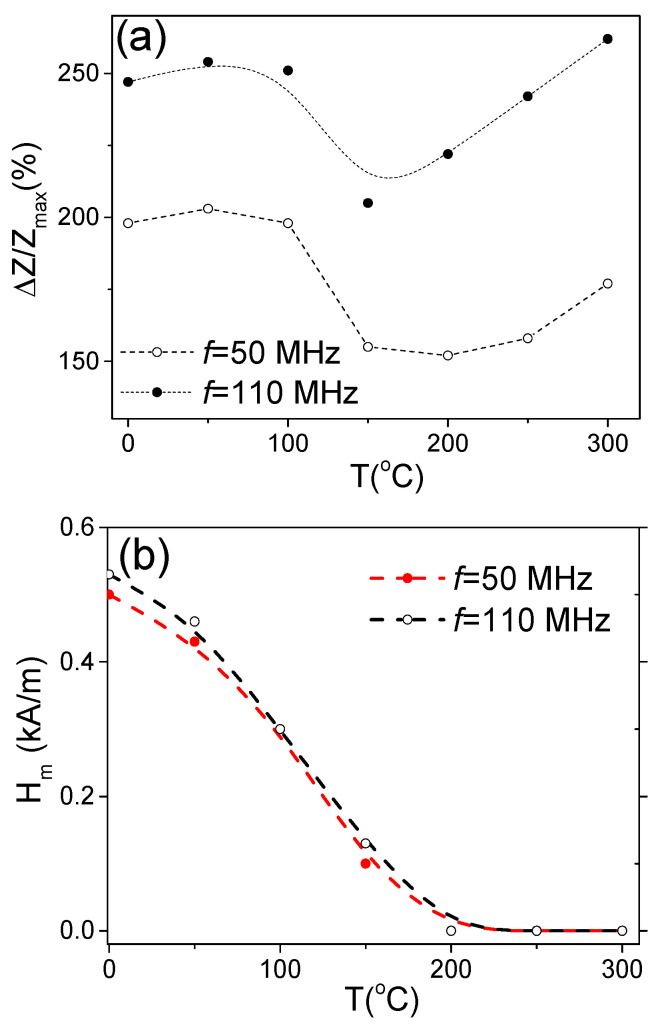
Δ*Z*/*Z_max_*(*T*) (**a**) and *H_m_*(*T*) (**b**) dependencies evaluated from Δ*Z*/*Z*(*H*) dependencies at 50 and 110 MHz in Co_69.2_Fe_3.6_Ni_1_B_12.5_Si_11_Mo_1.5_C_1.2_ sample.

The aforementioned features of the Δ*Z*/*Z*(*H*) dependencies are summarized in Figure 13, where the dependencies of Δ*Z*/*Z_max_* and *H_m_* versus temperature are provided. A decrease in Δ*Z*/*Z_max_* values between 150 and 200 °C is followed by an increase in Δ*Z*/*Z_max_* values at *T* ≥ 250 °C (see Figure 13a). Generally, a decrease in Δ*Z*/*Z_max_* values can be associated with the lower magnetic permeability of microwires with rectangular hysteresis loops. Typically, a lower GMI effect is reported elsewhere for magnetic wires with rectangular hysteresis loops [46,52]. However, the Δ*Z*/*Z_max_* values obtained at *T* = 300 °C (for *f* = 110 MHz) are even higher than for room *T* (see Figure 13). Accordingly, similarly to that discussed above for the case of Fe-rich microwires, an increase in Δ*Z*/*Z_max_* values at *T* ≥ 250 °C can be attributed to the Hopkinson effect. Such an interpretation looks reasonable, since the Curie temperature *T_c_* of the studied Co_69.2_Fe_3.6_Ni_1_B_12.5_Si_11_Mo_1.5_C_1.2_ sample is about 325 °C [54,55].

A gradual decrease in *H_m_* upon heating reflects the change in both hysteresis loops and Δ*Z*/*Z*(*H*) dependencies upon heating associated with the magnetic anisotropy change from transverse to axial (see Figure 13b).

Accordingly, similarly to the case of Fe-rich microwire, three main factors that affect the temperature dependence of hysteresis loops and the GMI effect must be underlined: (i) the Hopkinson effect, (ii) internal stresses’ relaxation, and (iii) temperature dependence of the internal stresses.

As mentioned above, the Hopkinson effect is characterized by a sharp maximum in magnetic permeability at temperatures slightly below *T_c_* [50,51,56,57]. Therefore, an increase in Δ*Z*/*Z_max_* and a decrease in *H_m_* observed in both of the studied samples at *T* ≥ 250 °C (see Figure 3l, Figure 7 and Figure 13a,b) can be attributed to the Hopkinson effect.

The difference between the influence of the internal stresses’ relaxation and the temperature dependence of internal stresses is that in the latter case the changes are reversible, while the internal stresses’ relaxation produces irreversible changes in magnetic properties.

**Figure 14:**
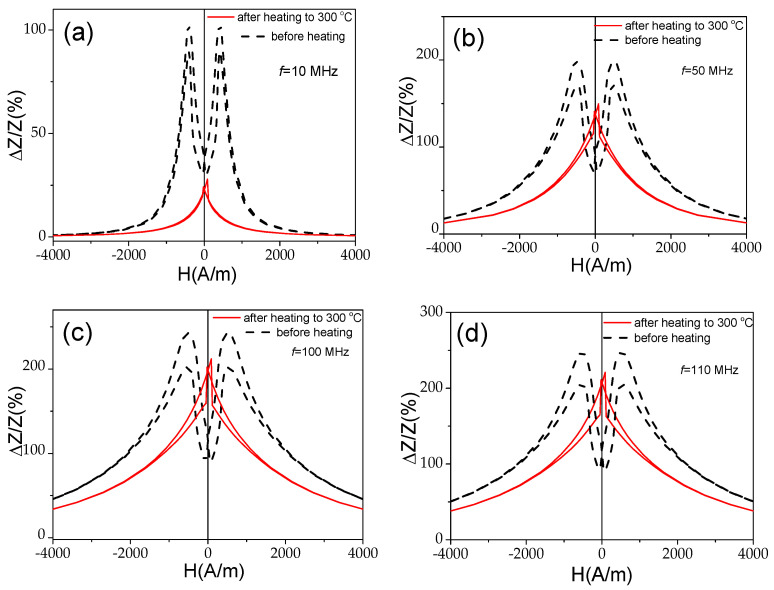
Δ*Z*/*Z*(*H*) dependencies measured at *f* = 10 MHz (**a**), 50 MHz (**b**), 100 MHz (**c**), and 110 MHz (**d**) in Co_69.2_Fe_3.6_Ni_1_B_12.5_Si_11_Mo_1.5_C_1.2_ sample before and after heating to 300 °C.

In the case of the studied Co_69.2_Fe_3.6_Ni_1_B_12.5_Si_11_Mo_1.5_C_1.2_ sample, the contribution of the internal stresses’ relaxation upon heating looks more relevant than for the Fe_75_B_9_Si_12_C_4_ sample microwire. This contribution is evidenced by an irreversible change in the Δ*Z*/*Z*(*H*) dependencies and Δ*Z*/*Z_max_* values of the studied Co-rich microwire after heating to 300 °C, followed by its cooling to room temperature (see Figure 14). Both the Δ*Z*/*Z*(*H*) dependencies and Δ*Z*/*Z_max_* values of the studied Co-rich microwire are substantially affected by heating up to 300 °C.

A change in Δ*Z*/*Z*(*H*) dependencies from double-peak to single-peak Δ*Z*/*Z*(*H*) dependence is observed after heating to 300 °C at all the measured frequencies (see Figure 14a,b). Additionally, a substantial decrease in Δ*Z*/*Z_max_* values is observed. This decrease is more noticeable at f = 10 MHz (see Figure 14a).

**Figure 15:**
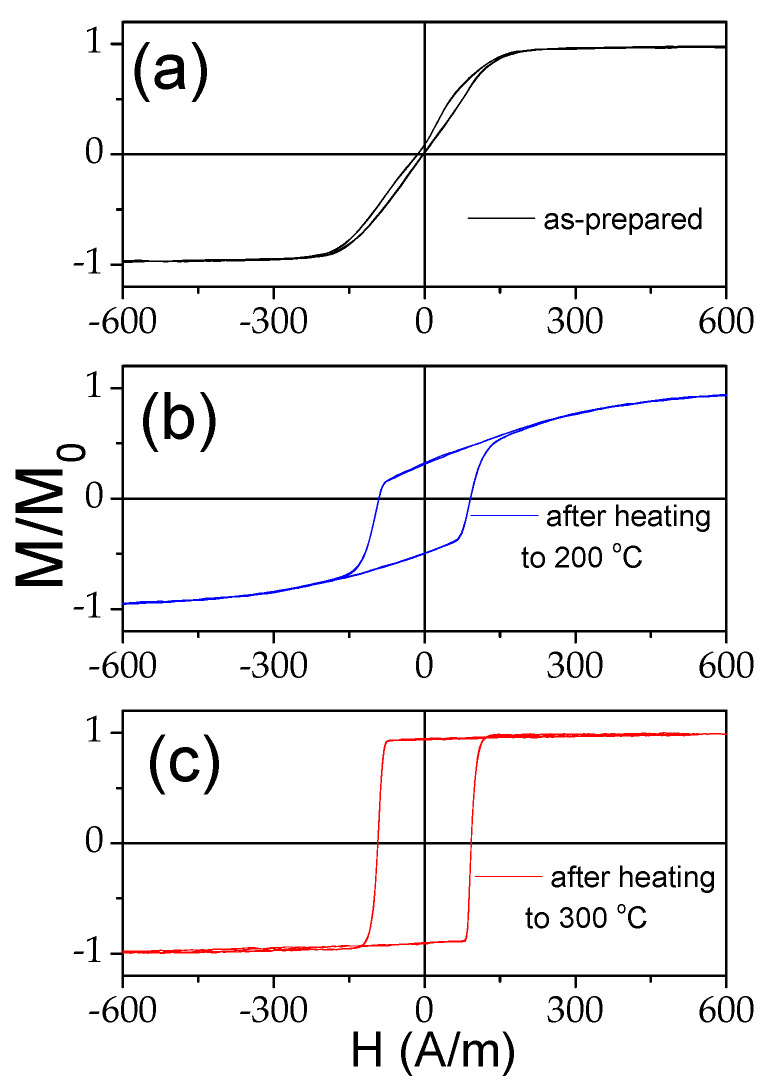
Hysteresis loops measured at room temperature of Co_69.2_Fe_3.6_Ni_1_B_12.5_Si_11_Mo_1.5_C_1.2_ sample before (**a**) and after heating to 200 °C (**b**) and 300 °C (**c**).

As discussed before, the single-peak Δ*Z*/*Z*(*H*) dependence is theoretically predicted and experimentally confirmed for magnetic wires with an axial character of magnetic anisotropy [45,46,53]. Therefore, the observed irreversible changes in Δ*Z*/*Z*(*H*) dependencies after heating must be attributed to the magnetic anisotropy changes after heating. The hysteresis loops measured at room temperature after heating to 200 °C and 300 °C are provided in Figure 15. Observed substantial changes after heating must be attributed to the change in the magnetic anisotropy from weak transverse magnetic anisotropy to axial magnetic anisotropy after heating.

It must be noticed that a modification of hysteresis loops in various Co-rich microwires from linear to rectangular upon annealing and even upon heating was recently observed and interpreted considering the change in the magnetostriction coefficient value and even sign upon annealing due to the stress dependence of the magnetostriction [38,58,59]. The relevant influence of internal stresses’ relaxation on the magnetostriction coefficient value and sign in Co-rich amorphous alloys with vanishing magnetostriction was reported and discussed in terms of the changes in the local atomic environment, clustering, and the internal stresses’ relaxation [58,59,60].

Accordingly, in contrast to the Fe-rich sample, the contribution of the irreversible changes in Δ*Z*/*Z*(*H*) dependencies related to stresses relaxation is more relevant in Co-rich microwire. Therefore, previous annealing of Co-rich microwires allowing internal stresses’ relaxation can be considered to avoid the observed irreversibility in Δ*Z*/*Z*(*H*) dependencies upon heating. Recently, such an assumption has been confirmed by measurements of the temperature dependence of the GMI effect in Co-rich microwires with stress annealing-induced magnetic anisotropy; in such microwires, the irreversibility in the GMI effect and hysteresis loops upon heating was substantially reduced [60].

In both of the studied microwires, the Δ*Z*/*Z_max_*(*T*) dependencies present similar features: a decrease in Δ*Z*/*Z_max_* at *T* ≈ 200 °C followed by an increase in Δ*Z*/*Z_max_* with *T* increasing (see Figure 7 and Figure 13a). To explain the observed similarity in the Δ*Z*/*Z_max_*(*T*) dependencies for both of the studied microwires, it is necessary to recall that a high GMI effect can be realized if δ is substantially affected by H through the µφ(*H*) dependence (see Equation (3)). It is generally accepted that the most favorable conditions for the implementation of the GMI effect are realized in magnetic wires with low transverse magnetic anisotropy and high magnetic permeability. Therefore, in both extreme cases, Co-rich microwires with axial magnetic anisotropy or Fe-rich wires with high transverse magnetic anisotropy, a high GMI effect is not expected. As discussed above, the Hopkinson effect is linked to a sharp magnetic permeability maximum at temperatures slightly below the Curie temperature, *T_c_*. In both studied microwires, a decrease in *H_k_* upon heating (at *T* ≥ 200–250 °C) is observed (see Figure 3c and Figure 9). Therefore, an increase in Δ*Z*/*Z_max_* at temperatures close to the Curie temperature, i.e., at *T* = 300 °C, must be attributed to the Hopkinson effect.

Finally, heating leads to both relaxation of internal stresses, *σ*_i_, and a change in *σ*_i_ due to their temperature dependence.

As discussed above, a linear *σ_i_*(*T*) dependence is expected in glass-coated microwires (given by Equation (3)) [33]. In the case of the studied Fe-rich microwire, the linear *Hk*(*T*) observed at *T* ≤ 150 °C can be related to the *σ_i_*(*T*) dependence. Below, we will try to evaluate the contribution linked with the change in internal stresses due to their temperature dependence in the studied Co-rich microwire.

While the applied stress dependence of hysteresis loops of amorphous microwires with positive *λ_s_* is studied in detail [26,27,61], there are only very few previous studies of the dependence of hysteresis loops and the magnetic anisotropy field, *H_k_*, of Co-rich microwires with inclined hysteresis loops on applied tensile stress, *σ* [28]. As experimentally demonstrated [28], the *H_k_*(*σ*) dependence of Co-rich microwires with inclined hysteresis loops has been well described by a linear increase in *H_k_* with *σ*. The origin of such linear *H_k_*(*σ*) dependence was attributed to the *λ_s_*(*σ*) dependence, previously reported in Co-rich wires with low and negative *λ_s_*, given as follows [58,59]:

(5) $$\lambda_{S,\sigma }=\lambda_{S,0}-B\sigma$$

*λ_s,o_*—the magnetostriction coefficient at *σ* = 0, *λ_s,σ_*—the magnetostriction coefficient at a given *σ*, and *B*—the positive coefficient of order 10^−10^ MPa.

Therefore, a linear decrease in *H_k_*(*T*) is expected for the contribution linked to the temperature dependence of the internal stresses.

**Figure 16:**
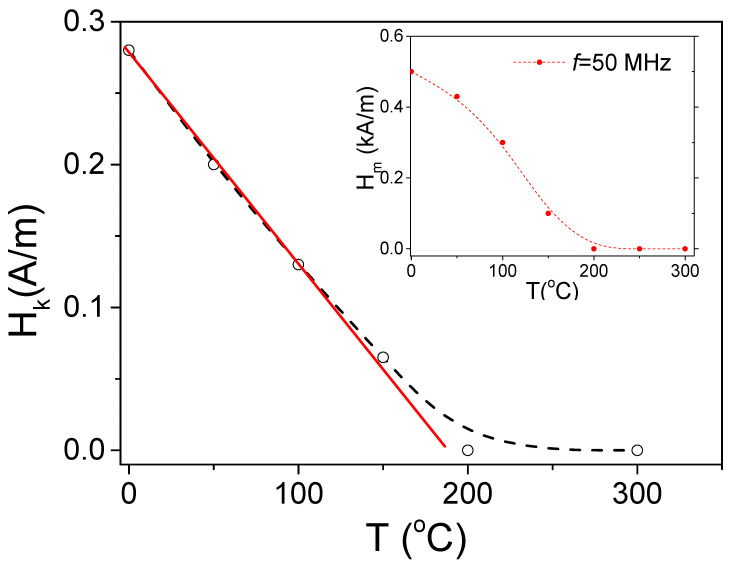
*H_k_*(*T*) dependence evaluated from hysteresis loops of Co_69.2_Fe_3.6_Ni_1_B_12.5_Si_11_Mo_1.5_C_1.2_ sample. *H_m_*(*T*) dependence is provided in the inset.

Similarly to what is provided in Figure 3l for the Fe_75_B_9_Si_12_C_4_ sample, we tried to evaluate *H_k_*(*T*) dependence for the Co_69.2_Fe_3.6_Ni_1_B_12.5_Si_11_Mo_1.5_C_1.2_ sample from the hysteresis loops shown in Figure 9. As can be appreciated from Figure 16, a linear *H_k_*(*T*) dependence can be roughly observed for *T* ≤ 150 °C. It is worth noting that the same tendency (linear *H_k_*(*T*) dependence) is observed for the Fe_75_B_9_Si_12_C_4_ sample at the same temperature range (see Figure 3l). Therefore, we can propose the following interpretation for the observed temperature dependencies of hysteresis loops and the GMI ratio in both of the studied samples:

The main contribution of the temperature dependence of internal stresses is at *T* ≤ 150 °C. In this temperature range, the changes in magnetic properties are reversible.

There is a noticeable contribution of the internal stresses’ relaxation at *T* ≥ 150 °C. This process is associated with irreversible changes in magnetic properties.

There is an improvement in the GMI affect at *T* ≥ 250 °C due to the Hopkinson effect.

Regarding the *H_k_*(*T*) dependence for the Co_69.2_Fe_3.6_Ni_1_B_12.5_Si_11_Mo_1.5_C_1.2_ sample, for comparison in the inset of Figure 16, we provided *H_m_*(*T*) dependence for the same sample evaluated from the Δ*Z*/*Z*(*H*) dependence. Such a comparison is provided considering that the field of maximum, *H_m_*, observed in the Δ*Z*/*Z*(*H*) dependencies is commonly attributed to the magnetic anisotropy field at MHz frequencies [44,53]. Generally, both *H_k_*(*T*) and *H_m_*(*T*) dependencies have the same trend. However, substantially higher (almost twice) *H_m_* values are observed (see Figure 16). Such difference in *H_k_* and *H_m_* values was previously discussed in terms of rather different magnetic anisotropy in the surface layer and in the bulk layer due to the interface layer between the metallic nucleus and glass coating [38]. On the other hand, the origin of the GMI effect at elevated frequencies was discussed in terms of ferromagnetic resonance, FMR [62,63], and higher *H_m_* values are expected.

Accordingly, the observed thermal dependence of the GMI effect in the studied microwires must be attributed to the interplay of the Hopkinson effect, relaxation of the internal stresses, temperature dependence of internal stresses, and related change in the magnetostriction coefficient.

## 4. Conclusions

We have observed the substantial influence of heating on the magnetic properties and giant magnetoimpedance, GMI, effect in Fe-rich and Co-rich glass-coated microwires with positive and vanishing magnetostriction, respectively.

In the Fe_75_B_9_Si_12_C_4_ microwire, a remarkable increase in the GMI effect and almost completely reversible change in hysteresis loop shape from rectangular to inclined were observed upon heating. At temperatures above 150 °C, spontaneous magnetic bistability disappears; however, after cooling to room temperature, the hysteresis loop restores its rectangular shape. In the Fe_75_B_9_Si_12_C_4_ microwire, the main contribution to the increase in Δ*Z*/*Z_max_* is related to the heating itself, since the Δ*Z*/*Z_max_* values obtained at *T* = 300 °C are almost twice as high.

Accordingly, the observed temperature dependence of the GMI effect and magnetic properties of Fe-rich microwires can be useful for temperature monitoring. However, the effect of heating must be separated from the internal stresses’ relaxation upon heating.

In the Co_69.2_Fe_3.6_Ni_1_B_12.5_Si_11_Mo_1.5_C_1.2_ microwire, the hysteresis loop changed its shape from inclined to rectangular upon heating. The observed changes in hysteresis loops correlate with the modification of Δ*Z*/*Z*(*H*) dependencies from double-peak to single-peak and with the change in the value of the maximum GMI ratio. However, the effect of internal stresses on the GMI effect is more pronounced, and after heating to 300 °C, irreversible changes in the Δ*Z*/*Z*(*H*) dependencies and hysteresis loops are observed.

The origin of the observed influence of heating on hysteresis loops, Δ*Z*/*Z*(*H*) dependencies, and GMI ratio values is discussed, considering the contributions from the Hopkinson effect, the temperature dependencies of the magnetostriction coefficient, and the internal stresses, as well as the internal stresses’ relaxation.

## Data Availability

No new data were created or analyzed in this study. Data sharing is not applicable to this article.
